# Supplementary material for: Minimal presynaptic protein machinery governing diverse kinetics of calcium-evoked neurotransmitter release
Source: Nat Commun. 2024 Dec 30;15:10741. doi: 10.1038/s41467-024-54960-1 (PMC11685451; doi:10.1038/s41467-024-54960-1)
Supplement: Supplementary file 1 — Supplementary Information [file 41467_2024_54960_MOESM1_ESM.pdf]

SUPPLEMENTARY MATERIALS

MINIMAL PRESYNAPTIC PROTEIN MACHINERY GOVERNING DIVERSE KINETICS OF CALCIUM-EVOKED NEUROTRANSMITTER RELEASE.

Dipayan Bose[1,2*], Manindra Bera[1,3*], Christopher A. Norman[4,5], Yulia Timofeeva[5], Kirill E. Volynski[3,4] and Shyam S. Krishnakumar[1,2,4]

[1]Nanobiology Institute, Yale University, West Haven, USA; [2]Department of Neurology, School of Medicine, Yale University, New Haven, USA; [3]Department of Cell Biology, School of Medicine, Yale University, New Haven, USA; [4]Department of Clinical and Experimental Epilepsy, UCL Queen Square Institute of Neurology, London, UK; [5]Department of Computer Science, University of Warwick, Coventry, UK.

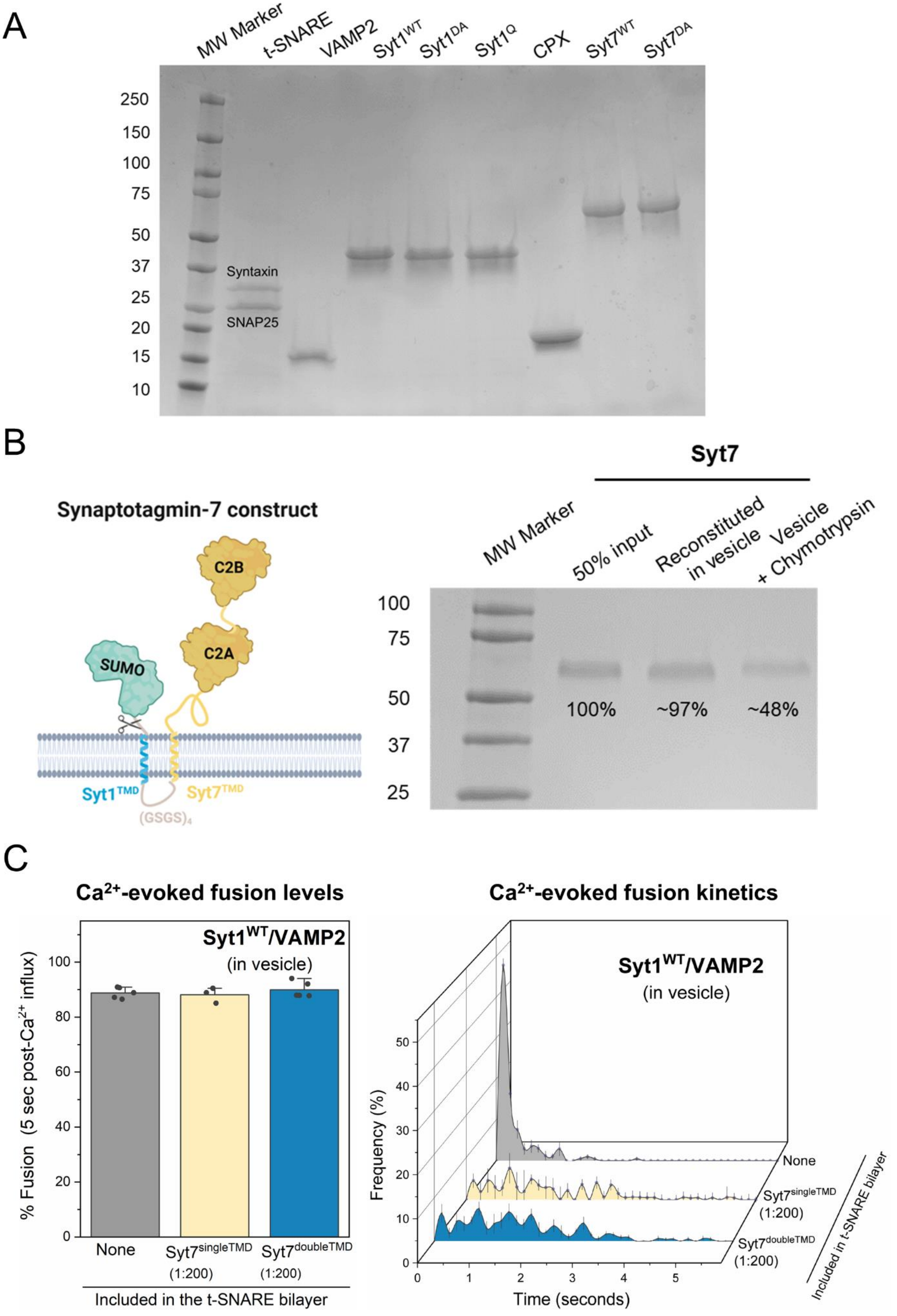
A
MW Marker
t-SNARE
VAMP2
Syt1WT
Syt1DA
Syt1Q
CPX
Syt7WT
Syt7DA
250
150
100
75
50
37
25
20
15
10
Syntaxin
SNAP25
B
Synaptotagmin-7 construct
C2B
C2A
SUMO
Syt1TMD
Syt7TMD
(GSGS)4
Syt7
MW Marker
50% input
Reconstituted in vesicle
Vesicle + Chymotrypsin
100
75
50
37
25
100%
~97%
~48%
C
Ca2+-evoked fusion levels
Syt1WT/VAMP2
(in vesicle)
% Fusion (5 sec post-Ca2+ influx)
100
80
60
40
20
0
None
Syt7singleTMD
(1:200)
Syt7doubleTMD
(1:200)
Included in the t-SNARE bilayer
Ca2+-evoked fusion kinetics
Syt1WT/VAMP2
(in vesicle)
Frequency (%)
50
40
30
20
10
0
None
Syt7singleTMD
(1:200)
Syt7doubleTMD
(1:200)
Included in t-SNARE bilayer
0
1
2
3
4
5
Time (seconds)

Supplementary Figure 1. (A) Coomassie stained SDS-PAGE gel image of the proteins used in the report. All proteins were expressed and purified using the bacterial expression system as described in the Methods section. (B) Left, Syt7 construct used in this study. The transmembrane domain (TMD) of Syt1 ($Syt1^{TMD}$) was included in addition to the $Syt7^{TMD}$ to enhance the reconstitution efficiency into the lipid membranes and GSGS linker ensured the proper orientation of $Syt7^{C2AB}$ domains. Right, Coomassie stained SDS-PAGE analysis of Syt7 reconsittuted in vesicles without or with Chymotrypsin treatment show ~50% of input Syt7 gets reconstituted into the vesicles with 50:50 inside:outside ratio. (C) In vitro functional analysis showed that the effects of the native Syt7 ($Syt7^{single\ TMD}$) and the Syt7 construct used in this study ($Syt7^{doubleTMD}$) on Ca^{2+}-evoked fusion are indistinguishable. Data (mean ± standard deviation) are from 3-5 independent experiments (N = 3 -5) for each condition (~ 40 – 50 vesicles per experiment). The source data is provided as a ‘Source Data’ file. Supplementary Figure 1B (left) created in BioRender. Krishnakumar, S. (2024) BioRender.com/x36q530.

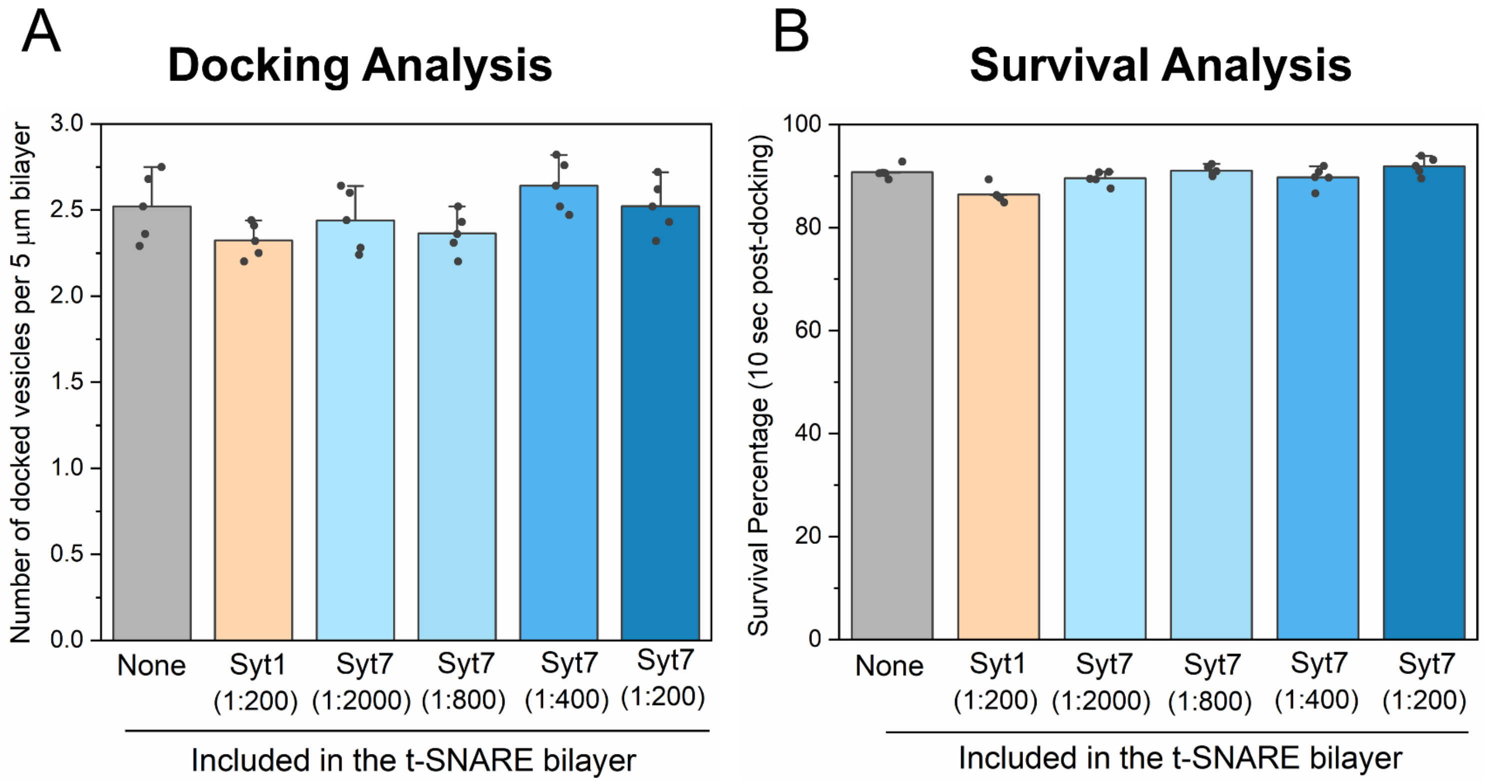

Supplementary Figure 2. Effect of Syt7 on the docking and fate of the docked vesicles under resting conditions (A) The inclusion of Syt7 in the bilayer did not affect the docking of the Syt1/VAMP2 vesicles, with a similar number of vesicles stably attaching to the bilayer under all conditions. (B) The majority (>90%) of the docked vesicle, irrespective of Syt7 presence, remained immobile and un-fused *i.e.*, stably 'clamped' at resting conditions. Data (mean ± standard deviation) are from 5 independent experiments (N = 5) for each condition (~ 40-50 vesicles per experiment). The source data is provided as a 'Source Data' file.

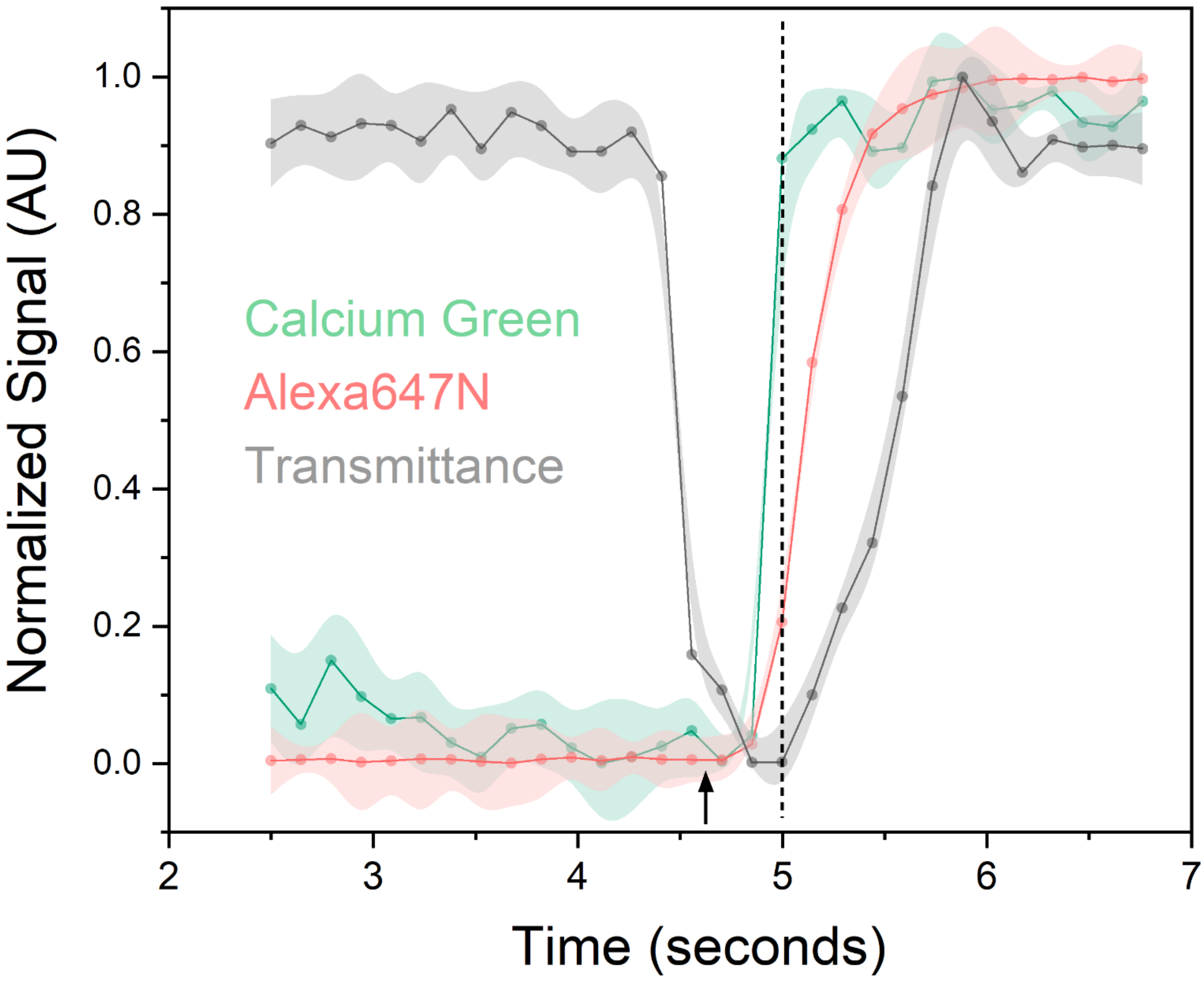

Supplementary Figure 3. To examine the Ca^{2+}-evoked fusion of docked vesicles, we added $CaCl_2$ (final [Ca^{2+}] of 100 μM in the experimental buffer) into the chamber, as denoted by the arrow. In all experiments, we used the transmittance signal to estimate the arrival of Ca^{2+} at the bilayer as it correlated with the minima of the transmittance signal (grey curve). For select cases, we also included a high-affinity Ca^{2+} sensor, Calcium Green C24 (green curve), in the bilayer to track the arrival of Ca^{2+} at the bilayer directly. We confirmed that it corresponds to the changes in the transmittance signal. Since Calcium Green fluorescence saturated within a single frame, in the calibration experiment, we used the soluble Alexa647 dye mixed with 100 μM $CaCl_2$ as a 'tracer' to track the actual diffusion of Ca^{2+} into the chamber (red curve) and thus estimate [Ca^{2+}] kinetics at docked vesicles. The source data is provided as a ‘Source Data’ file.

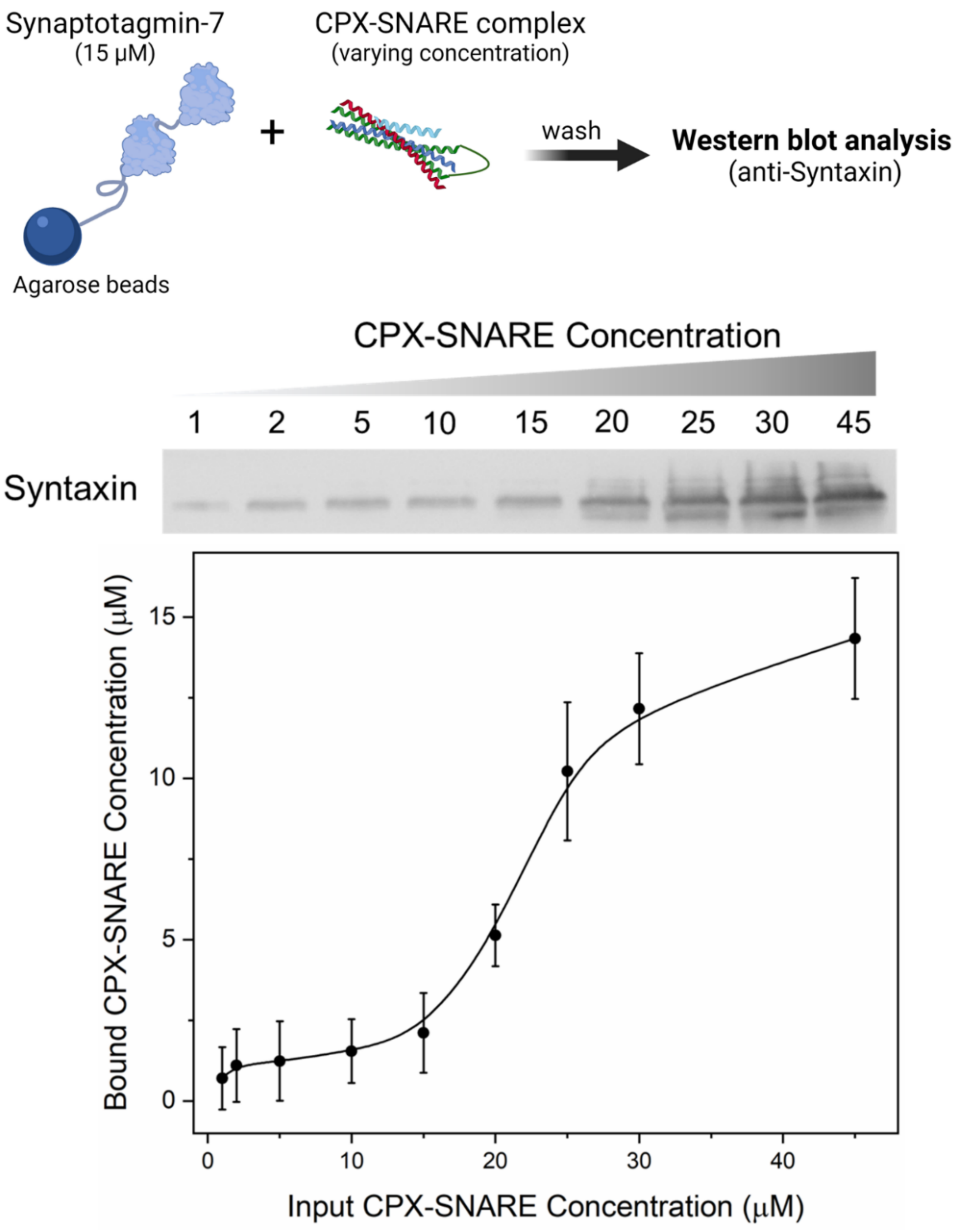

Supplementary Figure 4. Direct interaction between Syt7 and SNARE complex was examined using the pull-down assay with Syt7 as 'bait' and CPX-SNARE complex as 'prey'. The amount of SNARE bound was assessed using quantitative western-blot analysis with anti-Syntaxin antibody. We detected an increasing amount of CPX-SNARE bound to Syt7-containing agarose beads, with saturation observed around 30 μM input concentrations. Data from 3 independent pull-down experiments are shown. The source data is provided as a 'Source Data' file. Supplementary Figure 4 (top) created in BioRender. Krishnakumar, S. (2024) BioRender.com/c66g020.

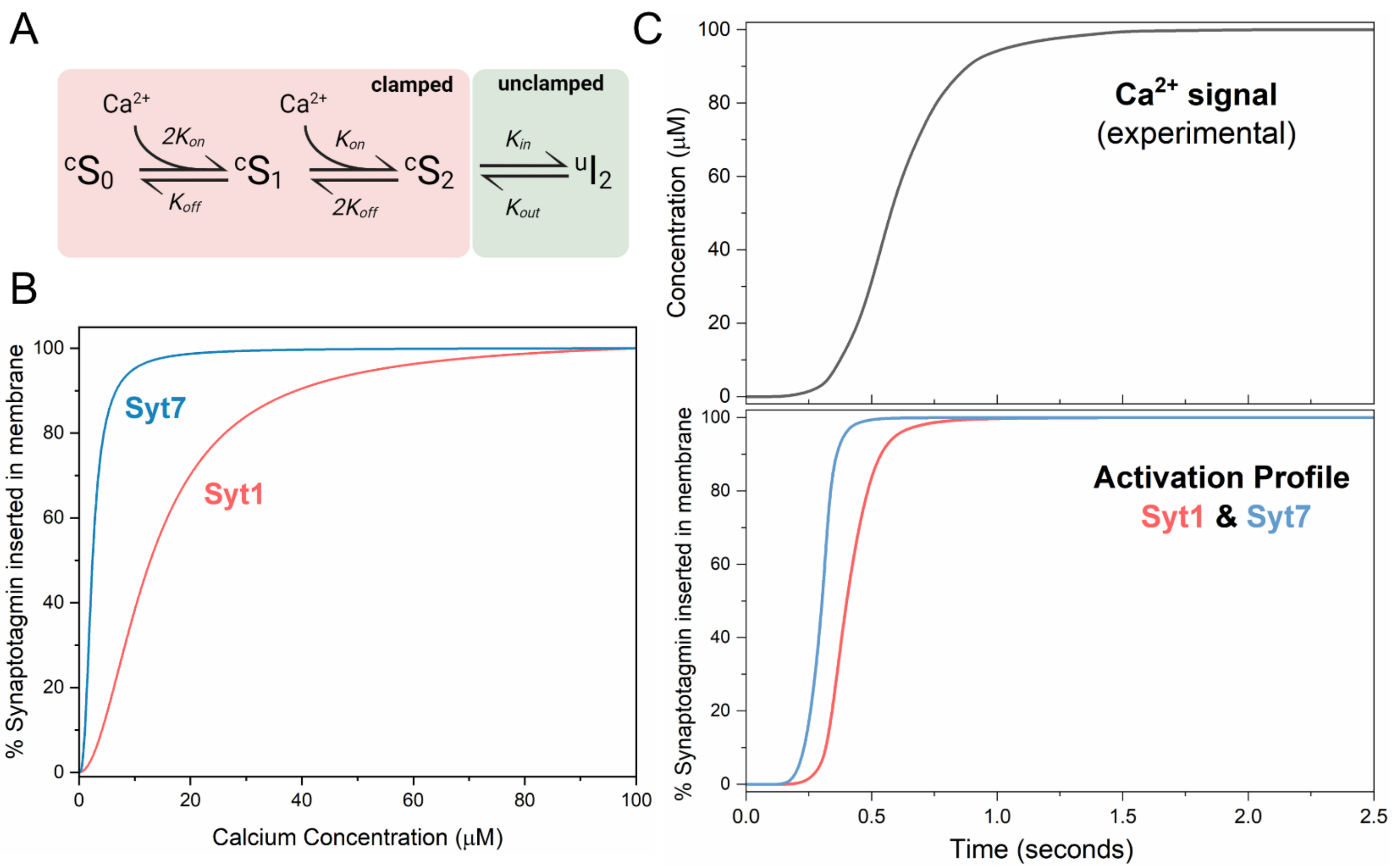

Supplementary Figure 5. Modeling of Ca^{2+}-dependent membrane insertion of Syt1 and Syt7 C2 domains under steady-state and experimental conditions. (A) Kinetic reaction scheme describing the Ca^{2+}-triggered membrane loop insertion of Syt1 and Syt7 C2 domains. The model parameters are outlined in the Methods section. (B) Consistent with available biochemical and biophysical data[1,2], model-predicted Ca^{2+} dependency for C2 domain membrane loop insertion under steady-state conditions shows a higher apparent affinity for Syt7 (K_d ~3 μM) and a lower apparent affinity for Syt1 (K_d ~13 μM). (C) Under conditions of a ramped Ca^{2+} increase to a saturating concentration of 100 μM, as in our experimental setup, Syt1 and Syt7 display comparable activation patterns for Ca^{2+}-dependent membrane insertion, with Syt7 being only slightly more sensitive than Syt1. The source data is provided as a ‘Source Data’ file.

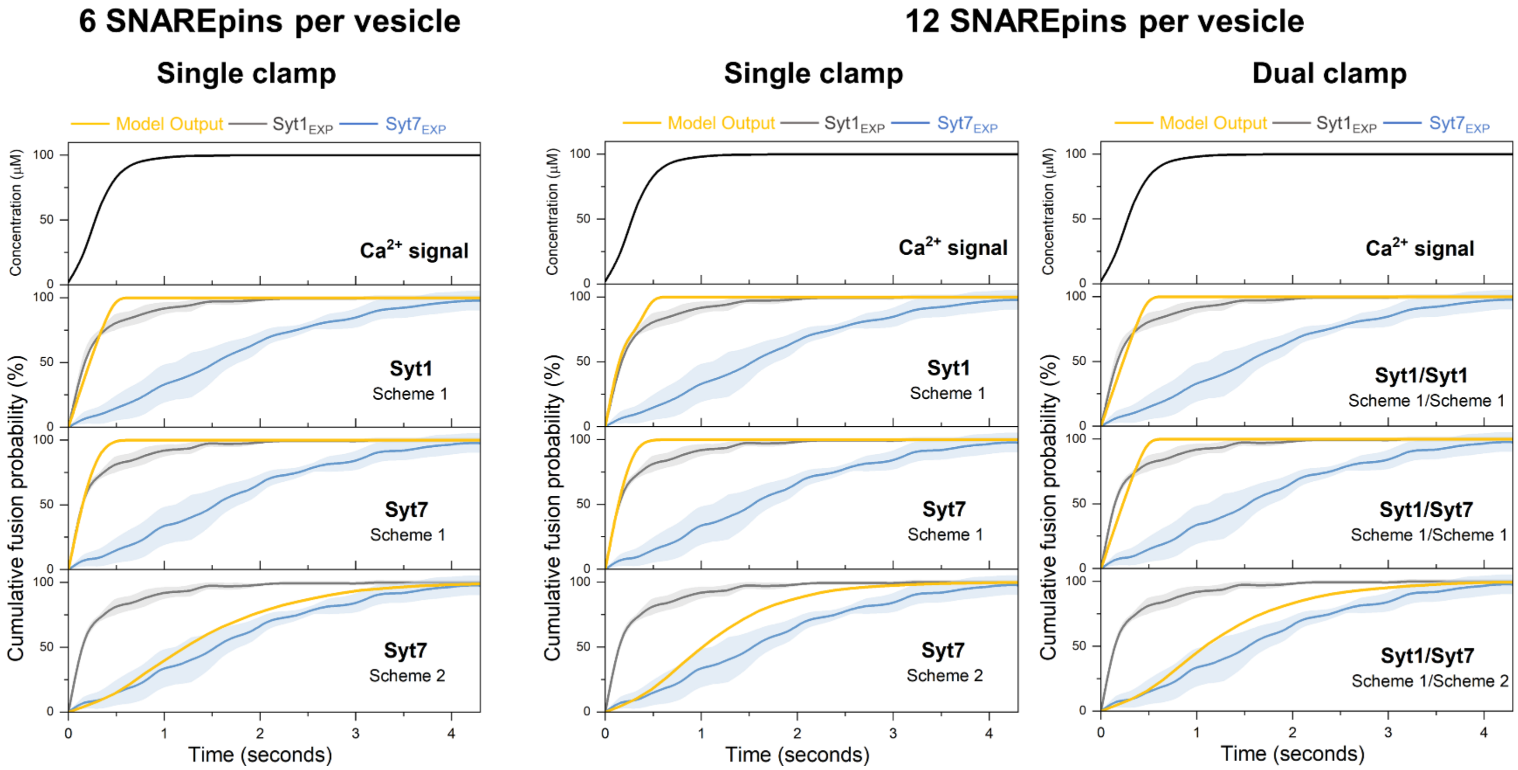

Supplementary Figure 6. The time course of vesicular fusion (Model Output) was simulated in response to the experimentally constrained Ca^{2+} signal (Supplementary Figure 3) for models with different numbers of SNAREpins and clamp architecture. As illustrated in Figure 4, we tested different kinetics of clamp reversal with Scheme 1 assuming instantaneous removal of the Synaptotagmin fusion clamp upon membrane insertion, and Scheme 2 assuming a delay. Experimental data (mean ± standard deviation from Figure 1C) for the Ca^{2+}-triggered fusion of Syt1 containing vesicles in the absence ($Syt1_{EXP}$) or the presence of saturating levels of Syt7 ($Syt7_{EXP}$) are plotted for comparison. The model suggests that observed fusion kinetics can be explained by the mechanism with differential rates of fusion clamp removal for Syt1 (instantaneous) and Syt7 (delayed), regardless of the number of SNAREpins per vesicle or the single vs. dual synaptotagmin fusion clamp architecture. For each modeled condition a minimum of 1000 stochastic simulations were performed to compute the average response. The source data is provided as a 'Source Data' file.

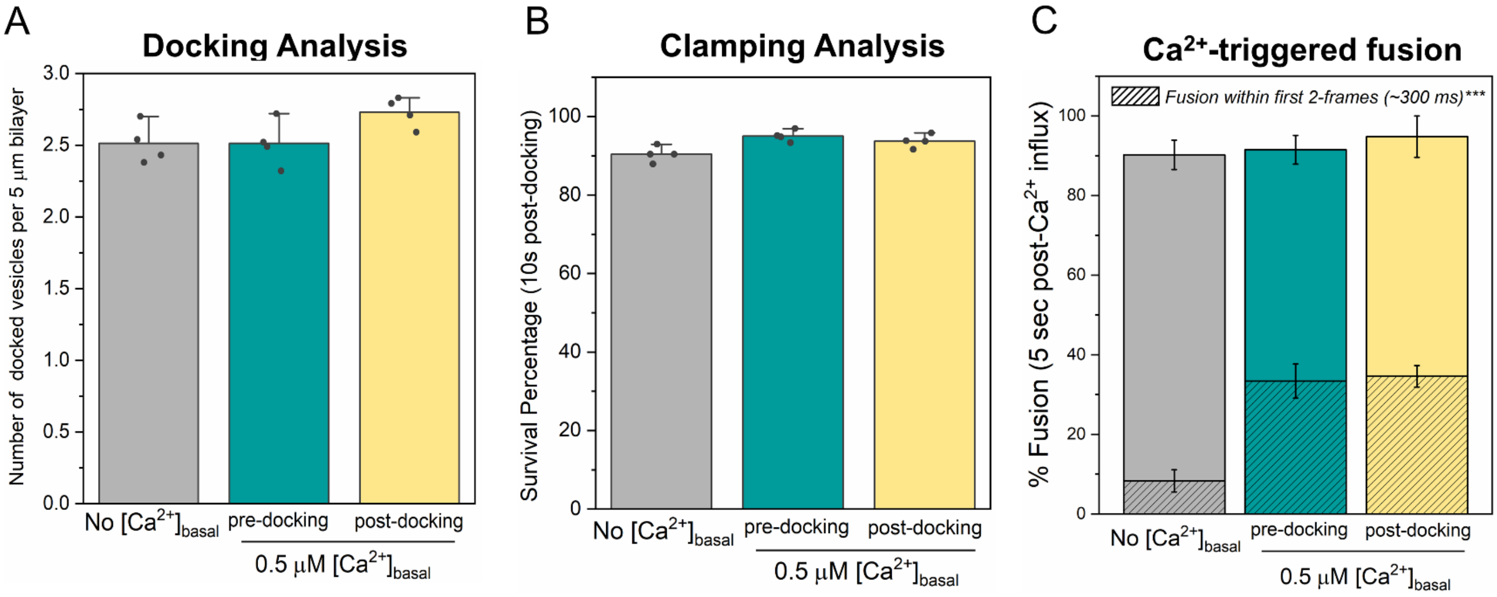

Supplementary Figure 7. The effect of elevated $[Ca^{2+}]_{basal}$ and the order-of-addition on vesicle docking, clamping, and Ca^{2+}-evoked fusion under $Syt1^{WT}$/$Syt7^{WT}$ conditions. (A, B) Increasing the $[Ca^{2+}]_{basal}$ to 0.5 μM during (green) or after (yellow) docking of the vesicles had no effect on number of vesicles docking or the fate of the docked vesicles as compared to the control (grey) condition with zero $[Ca^{2+}]_{basal}$. Similar number of docked vesicles were observed, and the majority remained stably clamped. (C) Elevating the $[Ca^{2+}]_{basal}$ enhanced the Ca^{2+}-evoked synchronous fusion component (hatch portion), with no observed difference based on order-of-addition of 0.5 μM $[Ca^{2+}]_{basal}$. This data demonstrates that Syt7 facilitates synchronous fusion of docked vesicles. All experiments were conducted using Syt1/VAMP2 vesicles, with Syt7 (1:200) included in the t-SNARE containing suspended bilayer. Data (mean ± standard deviation) are from 4 independent experiments (N = 4) for each condition (~ 40 – 50 vesicles per experiment). One-way ANOVA revealed statistically significant difference (***$p<0.001$) in Ca^{2+}-coupled fusion occurring within ~300 ms (hatched bar) between groups. The data from ANOVA and Tukey's HSD post-hoc comparing specific groups are shown in Supplementary Table 5. The source data is provided as a 'Source Data' file.

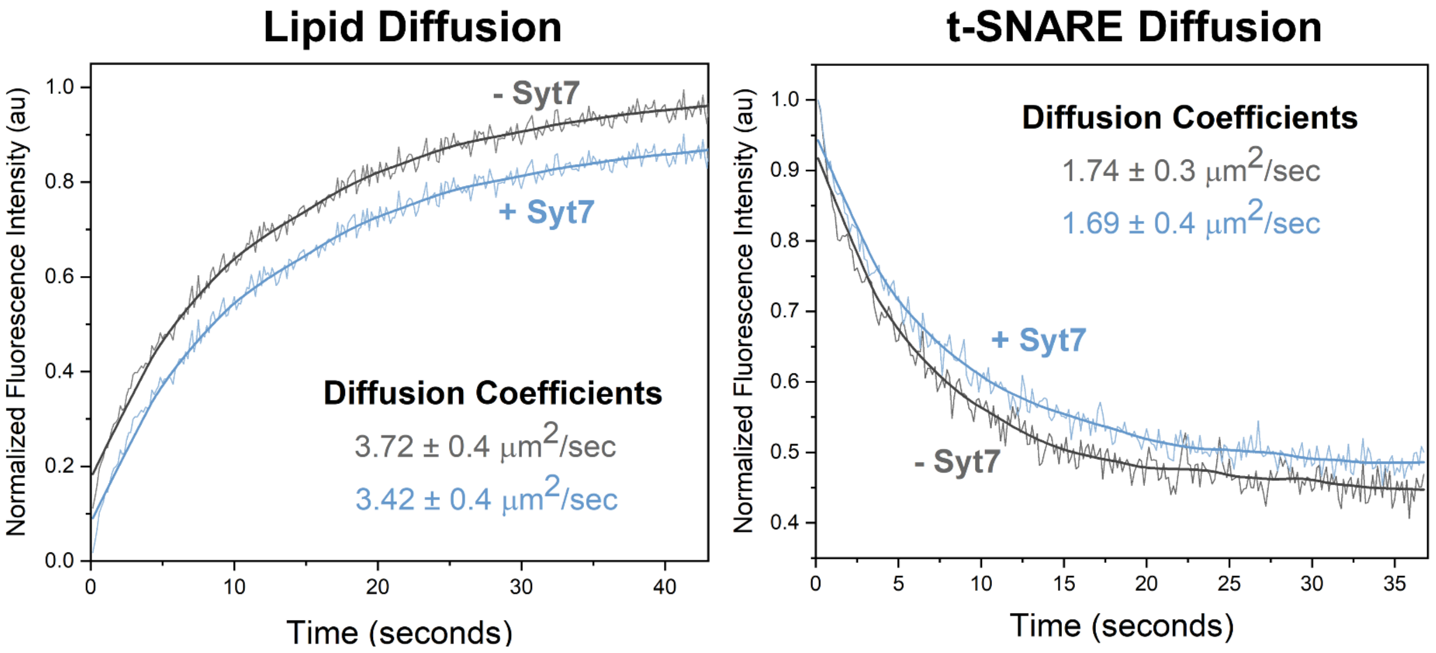

Supplementary Figure 8. The diffusion of lipid and t-SNARE on the suspended lipid bilayer is not affected by the inclusion of Syt7. The fluidity of the suspended lipid bilayer containing t-SNARE (Syntaxin/SNAP25) without or with Syt7 (1:200 protein-to-lipid ratio) was measured using fluorescence recovery after bleaching (FRAP) analysis and was analyzed on Wolfram Mathematica using a custom script to estimate the diffusion coefficient as described previously[3,4]. We used the fluorescence signal from ATTO465-PE (2%) included in the bilayer for lipid diffusion and Alexa568-labeled t-SNAREs reconstituted on the bilayer to track the protein diffusion. The source data is provided as a 'Source Data' file.

Supplementary Table 1. Statistical analysis for data in Figure 1B.

One-way ANOVA analysis

Source of Variation	*SS*	*df*	*MS*	*F*	*P-value*	F_{crit}
Between Groups	16194.01	5	3238.80	79.23	4.03556E-14	2.62
Within Groups	981.08	24	40.88			
Total	17175.09	29				

Tukey HSD analysis

Treatment pair	Q-statistic	p-value	Inference
A vs B	0.46	0.899	not significant
A vs C	5.10	<0.05	significant
A vs D	15.66	<0.01	significant
A vs E	16.99	<0.01	significant
A vs F	20.47	<0.01	significant
B vs C	5.56	<0.01	significant
B vs D	16.13	<0.01	significant
B vs E	17.45	<0.01	significant
B vs F	20.93	<0.01	significant
C vs D	10.56	<0.01	significant
C vs E	11.89	<0.01	significant
C vs F	15.37	<0.01	significant
D vs E	1.33	0.899	not significant
D vs F	4.80	<0.05	significant
E vs F	3.48	0.17	not significant

Conditions tested (i.e. protein included in the bilayer) ***A****: None;* ***B****: Syt1 (1:200);* ***C****: Syt7 (1:2000);* ***D****: Syt7 (1:800);* ***E****: Syt7 (1:400);* ***F****: Syt7 (1:200)*

Supplementary Table 2. Statistical analysis for data in Figure 2B.

One-way ANOVA analysis

Source of Variation	*SS*	*df*	*MS*	*F*	*P-value*	F_{crit}
Between Groups	15104.72	2	7552.36	1320.34	1.16506E-08	5.14
Within Groups	34.32	6	5.72			
Total	15139.04	8				

Tukey HSD analysis

Treatment pair	Q-statistic	p-value	inference
A vs B	62.57	<0.01	significant
A vs C	63.29	<0.01	significant
B vs C	0.72	0.86	not significant

Conditions tested (i.e. protein included in the bilayer) ***A****: None;* ***B****: Syt7 (1:800);* ***C****: Syt7 (1:200)*

Supplementary Table 3. Statistical analysis for data in Figure 3A.

One-way ANOVA analysis

Source of Variation	*SS*	*df*	*MS*	*F*	*P-value*	F_{crit}
Between Groups	12480.21	2	6240.10	276.90	9.10093E-11	3.89
Within Groups	270.42	12	22.54			
Total	12750.63	14				

Tukey HSD analysis

Treatment pair	Q-statistic	p-value	inference
A vs B	13.16	<0.01	significant
A vs C	33.05	<0.01	significant
B vs C	19.89	<0.01	significant

Conditions tested (i.e. protein included in the bilayer) ***A****: None;* ***B****: $Syt7^{DA}$ (1:800);* ***C****: $Syt7^{DA}$ (1:200)*

Supplementary Table 4. Statistical analysis for data in Figure 3B.

One-way ANOVA analysis

Source of Variation	*SS*	*df*	*MS*	*F*	*P-value*	F_{crit}
Between Groups	11755.83	2	5877.92	112.57	1.67887E-08	3.89
Within Groups	626.58	12	52.21			
Total	12382.41	14				

Tukey HSD analysis

Treatment pair	Q-statistic	p-value	inference
A vs B	9.98	<0.01	significant
A vs C	21.21	<0.01	significant
B vs C	11.23	<0.01	significant

Conditions tested (i.e. protein included in the bilayer) ***A****: None;* ***B****: Syt7 (1:800);* ***C****: Syt7 (1:200)*

Supplementary Table 5. Statistical analysis for data in Supplementary Figure 7C.

One-way ANOVA analysis

Source of Variation	*SS*	*df*	*MS*	*F*	*P-value*	F_{crit}
Between Groups	1770.93	2	885.46	118.34	3.44645E-07	4.26
Within Groups	67.34	9	7.48			
Total	1838.27	11				

Tukey HSD analysis

Treatment pair	Q-statistic	p-value	inference
A vs B	18.39	<0.01	significant
A vs C	19.26	<0.01	significant
B vs C	0.86	0.81	not significant

Conditions tested ($[Ca^{2+}]_{basal}$ added) ***A****: None;* ***B****: pre-docking;* ***C****: post-docking*

Supplementary References

1 Sugita, S., Shin, O. H., Han, W., Lao, Y. & Sudhof, T. C. Synaptotagmins form a hierarchy of exocytotic Ca(2+) sensors with distinct Ca(2+) affinities. *EMBO J* **21**, 270-280, doi:10.1093/emboj/21.3.270 (2002).

2 Voleti, R., Tomchick, D. R., Sudhof, T. C. & Rizo, J. Exceptionally tight membrane-binding may explain the key role of the synaptotagmin-7 C2A domain in asynchronous neurotransmitter release. *Proc Natl Acad Sci U S A* **114**, E8518-8527, doi:10.1073/pnas.1710708114 (2017).

3 Ramakrishnan, S., Bera, M., Coleman, J., Rothman, J. E. & Krishnakumar, S. S. Synergistic roles of Synaptotagmin-1 and complexin in calcium-regulated neuronal exocytosis. *Elife* **9**, doi:10.7554/eLife.54506 (2020).

4 Ramakrishnan, S. *et al.* High-Throughput Monitoring of Single Vesicle Fusion Using Freestanding Membranes and Automated Analysis. *Langmuir* **34**, 5849-5859, doi:10.1021/acs.langmuir.8b00116 (2018).
